# Identification of two novel autism genes, *TRPC4* and *SCFD2*, in Qatar simplex families through exome sequencing

**DOI:** 10.3389/fpsyt.2023.1251884

**Published:** 2023-10-31

**Authors:** Vijay Gupta, Afif Ben-Mahmoud, Bonsu Ku, Dinesh Velayutham, Zainab Jan, Abdi Yousef Aden, Ahmad Kubbar, Fouad Alshaban, Lawrence W. Stanton, Puthen Veettil Jithesh, Lawrence C. Layman, Hyung-Goo Kim

**Affiliations:** ^1^Neurological Disorder Research Center, Qatar Biomedical Research Institute (QBRI), Qatar Foundation, Hamad Bin Khalifa University (HBKU), Doha, Qatar; ^2^Disease Target Structure Research Center, Korea Research Institute of Bioscience and Biotechnology, Daejeon, Republic of Korea; ^3^College of Health & Life Sciences, Qatar Foundation, Hamad Bin Khalifa University (HBKU), Doha, Qatar; ^4^Section of Reproductive Endocrinology, Infertility and Genetics, Department of Obstetrics and Gynecology, Augusta University, Augusta, GA, United States; ^5^Department of Neuroscience and Regenerative Medicine, Augusta University, Augusta, GA, United States

**Keywords:** exome sequencing, autism, intellectual disability, digenic, TRPC4, SCFD2

## Abstract

This study investigated the genetic underpinnings of autism spectrum disorder (ASD) in a Middle Eastern cohort in Qatar using exome sequencing. The study identified six candidate autism genes in independent simplex families, including both four known and two novel autosomal dominant and autosomal recessive genes associated with ASD. The variants consisted primarily of *de novo* and homozygous missense and splice variants. Multiple individuals displayed more than one candidate variant, suggesting the potential involvement of digenic or oligogenic models. These variants were absent in the Genome Aggregation Database (gnomAD) and exhibited extremely low frequencies in the local control population dataset. Two novel autism genes, *TRPC4* and *SCFD2*, were discovered in two Qatari autism individuals. Furthermore, the D651A substitution in *CLCN3* and the splice acceptor variant in *DHX30* were identified as likely deleterious mutations. Protein modeling was utilized to evaluate the potential impact of three missense variants in *DEAF1*, *CLCN3*, and *SCFD2* on their respective structures and functions, which strongly supported the pathogenic natures of these variants. The presence of multiple *de novo* mutations across trios underscored the significant contribution of *de novo* mutations to the genetic etiology of ASD. Functional assays and further investigations are necessary to confirm the pathogenicity of the identified genes and determine their significance in ASD. Overall, this study sheds light on the genetic factors underlying ASD in Qatar and highlights the importance of considering diverse populations in ASD research.

## 1. Introduction

Autism spectrum disorder (ASD) is a highly heterogeneous neurodevelopmental disorder characterized by difficulties in social interaction and communication, restricted interests, and repetitive behaviors (1, 2). ASD is estimated to have a global prevalence of 1% among children, with a notably higher incidence in males, who are affected nearly four times more frequently than females (3). A recent survey conducted in the Middle Eastern Qatari population revealed a prevalence rate of 1 in 87 children between the ages of 6 and 11 (95% CI: 0.89–1.46) (4).

Individuals with ASD often experience co-occurring mental, neurological, or physical comorbidities such as intellectual disability (ID), seizures, sleep disturbances, craniofacial anomalies, and gastrointestinal issues, suggesting more complex genetic etiologies (5–10). Genetic factors play a significant role in ASD, with evidence of variants in numerous genes contributing to ASD risk (1, 11–15). The genetic underpinnings of ASD are extremely heterogeneous and involve many different genes, each of which is engaged in a variety of biological processes and pathways, including those that control synaptic plasticity, chromatin remodeling, gene transcription, and protein degradation (11, 16).

The genetic overlap between autism and intellectual disability is significant. An estimated 40 to 61% of individuals with ASD have coexisting intellectual disability (17). Current diagnostic laboratories utilize ID gene panels that consist of over 1,500 genes. Additionally, estimations suggest that approximately 10% of all 20,000 human genes could potentially be linked to intellectual disability (18). Consequently, even with a conservative estimate, it is reasonable to expect that up to 2,000 genes may be involved in autism.

Autism spectrum disorder has been associated with various genetic factors, including chromosomal structural variations, rare copy number variations (CNVs), and single nucleotide variants (SNV) (14, 19–22). Specifically, rare, hereditary, and *de novo* CNVs have been linked to a number of neurodevelopmental disorders, with approximately 15–20% of individuals with ASD exhibiting these variations (19, 21, 23). Despite the identification of a large number of ASD susceptibility genes, only a small number of them have undergone substantial validation (19). Consequently, the task of identifying individual causal genes remains challenging.

Recent advancements in next generation sequencing have revolutionized the process of gene discovery by generating a vast amount of data. A variety of methods have been employed to identify genetic variants associated with ASD, including exome sequencing (ES) and genome sequencing (GS). These techniques have proven instrumental in pinpointing rare and unique genetic variants associated with neurodevelopmental diseases, where ES was employed (24). However, genetic studies focusing on autism patients in Mid-Eastern countries are noticeably underrepresented. This gap in genetic investigations hinders the comprehensive understanding of ASD’s underlying mechanism within diverse populations and potentially delays the development of tailored interventions and treatments.

To this end, our study focused on ES and examined six families from Qatar, each with an autistic proband. The overarching goal was to gain deeper insights into the genetic underpinnings and pathophysiology of ASD. To achieve this, we designed an analysis pipeline specifically tailored to identify rare *de novo* and autosomal recessive variants in novel or previously known ASD genes. Protein modeling was utilized to evaluate how missense variants might affect the structure and functionality of our candidate genes. Furthermore, CADD scores, M-CAP, and ACMG guidelines were employed to predict, prioritize, and interpret the deleteriousness of genetic variants.

## 2. Results

**FIGURE 1:**
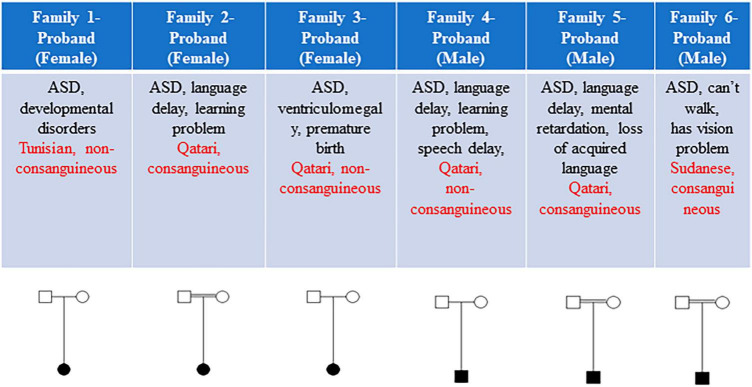
The clinical features and pedigrees of the six probands. Each subject’s sex, brief clinical features, ethnicity, and consanguinity are detailed. Three out of six families exhibited consanguinity.

This study included a total of six eligible participants, all displaying ASD, from six distinct families (Figure 1). Table 1 provides a comprehensive summary of the clinical features exhibited by these individuals, as well as the genetic variants identified. Common phenotypes found in all probands included autism and language/speech delay. One out of six individuals had vision problems, and two out of six individuals had loss of acquired language as additional phenotypes along with autistic features (Figure 1).

**TABLE 1:** Primary candidate genes and clinical features of six ASD subjects in Qatar.

Family ID	Family 1	Family 2	Family 3	Family 4	Family 5	Family 6
Consanguinity	−	+	−	−	+	+
Nationality	Tunisian	Qatari	Qatari	Qatari	Qatari	Sudanese
Age (Mom, Dad, Pro)	32, 38, 16	26, 60,30	27, NA, 16	43, 50, 14	NA, 57, 14	Na, 53, 18
Sex	F	F	F	M	M	M
Candidate Gene	*DEAF1* known	*CLCN3* known	*TRPC4* novel	*KMT2C* known	*SCFD2* novel	*DHX30* known
Chromosome	11p15.5	4q33	13q13.3	7q36.1	4q12	3p21.31
Nucleotide change	c.674G > T	c.1952A > C	c.379-2A > T splicing	c.2573G > T	c.1148C > T	c.1813-1G > T splicing
Accession number	NM_021008.4	NM_001243374.2	NM_016179.4	NM_170606.3	NM_152540.4	NM_014966.4
Genomic coordinate (hg19)	686,988	170,628,301	383,205,94	151,935,871	541,401,56	47,888,762
Exon/Intron	exon 5	exon 10	Splice site-intron 2	exon 15	exon 4	Splice site-intron 12
Effect on protein	p.G225V	p.D651A	Splicing	p.W858L	p.P383L	Splicing
Inheritance	*De novo*	*De novo*	*De novo*	*De novo*	Homozygous recessive	*De novo*
CADD	26.5	22.6	34	27	23	33
M-CAP	Possibly pathogenic	Possibly pathogenic	NA	NA	Possibly pathogenic	NA
ACMG	PS2, PS4, PM2, PP2, PP3 pathogenic	PS2, PS4, PM2, PP2, PP3 pathogenic	PVS1, PS2, PS4, PM2, PP3 pathogenic	PS2, PS4, PM2, PP2, PP3 pathogenic	PS4, PM2, PM3, PP3 likely pathogenic	PVS1, PS2, PS4, PM2, PP2, PP3 pathogenic
Allele frequency (gnomAD/QGP)	0/0.0001	0/0.00054	0/6.8e-05	0/0.0001	0.0046% (European)/0.13	0/0.0001
Autism	+	+	+	+	+	+
Developmental delay	−	−	+	−	+	+ walked independently at 1.5 years
Intellectual disability	−	n/a	n/a	+ mental retardation	+ mental retardation	n/a
Language/speech delay	+	+	n/a	+	+ delayed at 7 and 12 months	+
Loss of acquired language skills	+30 months	−	−	−	+	−
Loss of acquired motor skills	n/a	n/a	−	n/a	+	−
Learning problems	−	+	n/a	+	n/a	−
Loss of acquired social skills	+ 30 months	−	n/a	−	−	−
Behavior	+ aggressive	n/a	− normal	n/a	n/a	+ withdrawn
Head circumference	n/a	n/a	+ > 90%	n/a	n/a	n/a
Other clinical features	Loss of acquired skill		Preterm baby, chronic lung disease, VP shunt insertion, ventriculomegaly, widened cisterns, hypoplastic cerebellar hemisphere, small hypodense lesion at lacunar infarct + hydrocephalus, cortical blindness	Fragile-X-syndrome +	Prenatal complication, head trauma at 8 months	

“n/a” denotes not available, while (−) represents absence of the corresponding phenotype. QGP is allele frequency in Qatar Genome Program based on local population in Qatar. The age of each parent at the proband’s birth and the current age of the proband are denoted as follows: mother-Mom, father-Dad and proband-Pro where data is available (Age-Mom, Dad, Pro).

To identify SNVs, we conducted ES on each subject with both parents. This yielded approximately 40 Mbp of on target-reads, resulting in an average mean target region coverage of 80X for all samples. To narrow down the list of variants, we implemented a rigorous filtering approach. Specifically, we filtered out variants with a minor allele frequency (MAF) of more than 1% based on data from the Genome Aggregation Database (gnomAD) and Qatar Genome Programme databases. This step helped us to establish a link between the identified genotype and the general neurodevelopmental phenotype within each family.

**FIGURE 2:**
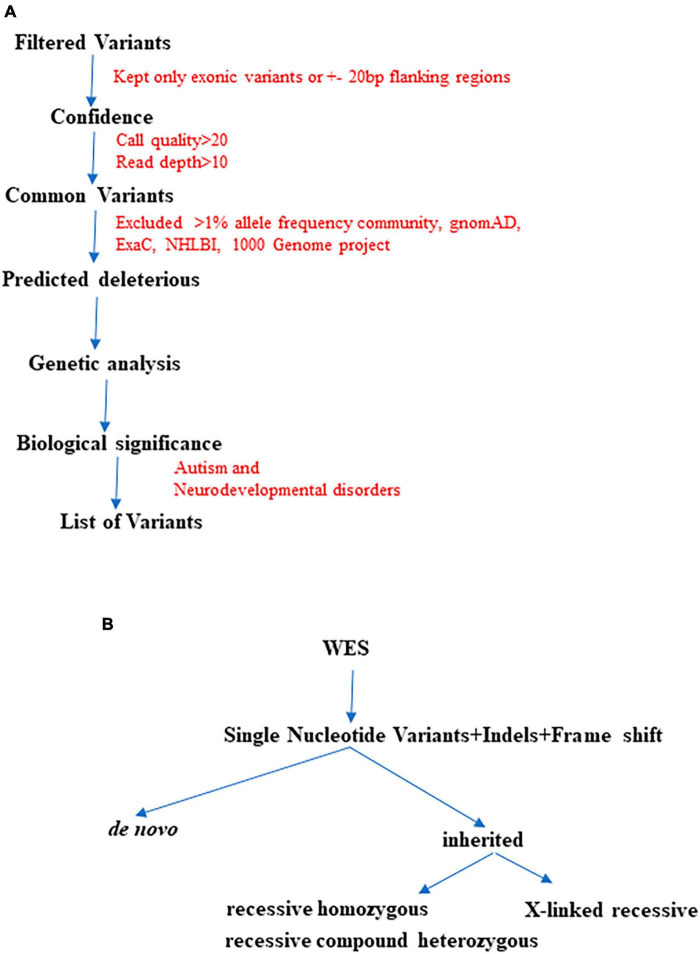
The finalization of variants involved utilizing multiple QCI-IT analysis parameters, as indicated in the workflow above **(A)**. Furthermore, during the final analysis, potential pathogenic variants were prioritized based on their inheritance patterns **(B)**.

Initially, we began with >50,000 variants per subject obtained from the original FASTQ files. However, we applied multiple filters such as confidence, predicted deleteriousness and genetic analysis. Through this process, we successfully reduced the variants to approximately <100 per subject. As illustrated in Figure 2, we further refined the variant selection by employing trio analysis and excluding common variants, which led to a significant reduction in the number of variants per subject. Ultimately, we were able to identify and retain approximately 4 variants per subject, whenever feasible.

We hypothesized that the six probands would possess either known or novel autosomal dominant heterozygous *de novo*, autosomal recessive homozygous, or X-linked recessive hemizygous candidate gene variants associated with ASD. To evaluate the conservation of the variants and predict their deleterious effects on gene function, several bioinformatics software’s were employed. Variants that resulted in obvious protein-altering changes (nonsense, insertions or deletions, missense, and splice sites) with a minor allele frequency of less than 1% were prioritized based on various protein prediction software (SIFT, PolyPhen-2, CADD, and M-CAP). To gain further insight into the harmful consequences at the protein level, several variants were modeled at the molecular level. The potential biological and clinical plausibility of gene functions and pathways were evaluated by thoroughly reviewing the literature and considering clinical phenotypes of the subjects. In total, we identified 13 candidate variants, consisting of nine *de novo* and four homozygous variants (Tables 1, 2). From these, one variant per subject was prioritized based on its high CADD score (>20), pathogenicity assessments using M-CAP and ACMG classification (Table 1). Notably, we did not find any X-linked variant in our six subjects.

**TABLE 2:** Secondary candidate genes and clinical features of five ASD subjects in Qatar.

Family ID	Family 1	Family 2	Family 3	Family 4	Family 6		
Consanguinity	−	+	−	−	+		
Nationality	Tunisian	Qatari	Qatari	Qatari	Sudanese		
Sex	F	F	F	M	M		
Candidate Gene	*EPHA5* novel	*LRIF1* novel	*RGS22* novel	*KMT5A* novel	*PARP14* novel	*PDPR* novel	*LRRC18* novel
Chromosome	4q13.1	1p13.3	8q22.2	12q24.31	3q21.1	16q22.1	10q11.23
Nucleotide change	c.680G > T	c.1240A > G	c.2386G > A	c.31C > T	c.2906C > G	c.697G > C	c.416_417delCAinsTG
Accession number	NM_182472.5	NM_018372.4	NM_001286693.2	NM_020382.7	NM_017554.3	NM_001322118.1	NM_001006939.4
Genomic coordinate (hg19)	66,467,589	111,494,266	101,011,510	123,874,000	122,420,307	70,166,203	501,217,84
Exon/Intron	exon 3	exon 2	exon 17	exon 2	exon 6	exon 7	exon 2
Effect on protein	p.R227L	p.I414V	p.A796T	p.R11C	p.A969G	p.E233Q	p.T139M
Inheritance	Homozygous recessive	*De novo*	*De novo*	*De novo*	Homozygous recessive	*De novo*	Homozygous recessive
CADD	28.5	20.6	23.4	20.5	22.6	25.3	25.4
M-CAP	Likely Benign	Likely Benign	Likely Benign	Possibly pathogenic	Possibly pathogenic	n/a	n/a
ACMG	PS4, PM2, PM3, PP3 likely pathogenic	PS2, PS4, PM2, PP3 pathogenic	PS2, PS4, PM2, PP3 pathogenic	PS2, PS4, PM2, PP3 pathogenic	PS4, PM2, PM3, PP3 likely pathogenic	PS2, PS4, PM2, PP3 pathogenic	PS4, PM2, PM3, PP3 likely pathogenic
Allele frequency (gnomAD/QGP)	0/0.0004	0/0.0003	0/0.0003	0/6.8e-04	0/3.4e-05	0/3.43-05	0/7.11e-05
Autism	+	+	+	+	+	+	+
Developmental delay	−	−	+	−	+ walked independently at 1.5 years	+ walked independently at 1.5 years	+ walked independently at 1.5 years
Intellectual disability	−	n/a	n/a	+	n/a	n/a	n/a
Language/speech delay	+	+	n/a	+	+	+	+
Loss of acquired language skills	+30 months	−	−	−	−	−	−
Learning problems	−	+	n/a	+	−	n/a	n/a
Loss of acquired social skills	+30 months	−	n/a	−	−	n/a	n/a
Behavior	+ aggressive	n/a	− normal	n/a	+ withdrawn	n/a	n/a
Head circumference	n/a	n/a	+ > 90%	n/a	n/a	n/a	n/a
Other clinical features	Loss of acquired skill		APGAR score 4 and 9 at 1 and 5 min, twin boy died at 3 h, frequent apnea	Fragile-X-syndrome +			

“n/a” denotes not available, while (−) represents absence of the corresponding phenotype. QGP is allele frequency in Qatar Genome Program based on local population in Qatar.

### 2.1. Identification of candidate variants

In subject 1 of family 1, we identified a *de novo* heterozygous missense SNV in the *DEAF1* transcription factor gene (MIM 602635) as shown in Table 1. *De novo* heterozygous mutations in *DEAF1* cause intellectual disability, speech impairment, and autistic behaviors (25). Multiple *de novo* heterozygous missense mutations have been identified in *DEAF1* causing Vulto-van Silfhout-de Vries syndrome characterized by ID with severe speech defects and behavioral problems (25–28). Additionally, homozygous recessive mutations in *DEAF1* have been identified in subjects with autism, hypotonia, language delay, ID, basal ganglia dysfunction, and epilepsy (29, 30). The variant c.674G > T (NM_021008.4) in exon 5 predicted a p.G225V substitution which was deemed deleterious, because this variant was not reported in gnomAD and highly disruptive by SIFT and Polyphen 2, respectively. The variant had a CADD score of 26.5 and was classified as pathogenic based on ACMG classification (PS2, PS4, PM2, PP2, PP3) and as possibly pathogenic by M-CAP (Table 1) (31). DEAF1 exhibits high levels of expression in the brain and central nervous system, implying its involvement in early neurodevelopmental stages (25, 30).

Within the same subject, we found an additional variant in *EPHA5* (EPH receptor A5, MIM 600004), a gene that has not been previously linked to neurodevelopmental disorders. Eph receptors including *EPHA5*, have been found to modulate the strength of existing synapses in the adult brain (32). The identified variant c.680G > T (NM_182472.5) was homozygous and caused p.R227L change by disrupting exon 3. It was predicted to be likely benign by M-CAP with CADD score 28.5 and classified as likely pathogenic by ACMG due to PS4, PM2, PM3, and PP3 (Table 2).

In family 2, we found two different *de novo* variants, in chloride voltage-gated channel 3, *CLCN3* (MIM 600580) and the ligand dependent nuclear receptor interacting factor 1, *LRIF1* (MIM 615354) genes. *De novo* heterozygous missense variants in *CLCN3* were identified in 9 unrelated patients who had neurodevelopmental disorders with hypotonia and brain abnormalities (33). *CLCN3* variant c.1952A > C (NM_001243374.2) at 4q33 in exon 10 in subject 2 resulted in p.D651A. This was a deleterious variant with CADD score 22.6 and classified as pathogenic by M-CAP and with ACMG classification of PS2, PS4, PM2, PP2, and PP3 (Table 1).

Another candidate gene *LRIF1*, located at 1p13.3, has been suggested as a potential neurodevelopmental candidate gene. Its physical interaction with four established neurodevelopmental genes (*PQBP1*, *CHD3*, *CHD4*, and *ADHC1*) further enhances its appeal as a candidate gene for ASD in this proband (34). The variant in *LRIF1* in subject 2 had c.1240A > G (NM_018372.4) change, resulting in p.I414V in exon 2. The CADD score of this variant was 20.6 and based on ACMG classification-PS2, PS4, PM2, PP3, it was denoted as pathogenic, and likely benign by M-CAP prediction (Table 2). A homozygous frameshift mutation in *LRIF1* was reported in a man with digenic facioscapulohumeral muscular dystrophy-3, who was born to consanguineous Japanese parents (35).

After considering factors such as the CADD score, M-CAP, and ACMG interpretation, *CLCN3* takes precedence over LRIF1 in terms of prioritization.

Within family 3, we identified two *de novo* variants in *TRPC4* (Transient receptor potential cation channel subfamily C member 4, MIM 603651) and *RGS22* (Regulator of G protein signaling 22, MIM 615650). Notably, neither of these genes has been previously reported to be associated with neurodevelopmental disorders. *TRPC4* located at 13q13.3 harbored a splicing variant c.379-2A > T (NM_016179.4), with a high CADD score of 34, indicating its deleterious nature. All splice sites at the exon/intron boundaries consistently features dinucleotides. GT at the 5′ splice donor site and AG at the 3′ splice acceptor site. Point mutations within these consensus sequences can disrupt splicing event, leading to the generation of an aberrant transcript from the affected gene (36). According to ACMG classification, this variant was classified as pathogenic, based on criteria PVS1, PS2, PS4, PM2, and PP3 (Table 1). Second candidate gene, *RGS22* at 8q22.2, on the other hand, is a member of a protein family involved in the regulation of G protein signaling pathway (37). Overexpression of *RGS22* has been shown to reduce cell migration and invasive potential in a metastatic esophageal cancer cell line (38). The *RGS22* variant in family 3 was c.2386G > A (NM_001286693.2) in exon 17, resulting in p.A796T. This variant was also deemed deleterious with a CADD score of 23.4 and classified as pathogenic according to ACMG classification criteria PS2, PS4, PM2, and PP3 (Table 2). *TRPC4* emerged as the prime candidate gene over *RGS22*, given its deleterious splicing variant and high CADD score of 34.

In family 4, we detected *de novo* variants in two lysine methyltransferases, *KMT2C* (Lysine methyltransferase 2C, MIM 606833) at 7q36.1 and KMT5A (Lysine methyltransferase 5A, MIM 607240) at 12q24. While *KMT5A* represents a novel gene, *KMT2C*, also known as *MLL3*, is a well-established gene linked to intellectual disability and autism (39). *KMT2C de novo* heterozygous mutations have been associated with severe intellectual disability in 3 unrelated patients (40). Knockdown of *KMT2C* in the drosophila ortholog “trr” impaired short-term memory in the mushroom body of the fly brain (41). The *KMT2C* variant was c.2573G > T (NM_170606.3) in exon 15, resulting in p.W858L. The variant was predicted to be deleterious and probably damaging due to a high CADD score of 27 and classified as pathogenic according to ACMG criteria PS2, PS4, PM2, PP2, and PP3 (Table 1). *KMT5A* (also known as *SETD8*) plays a role in transcriptional regulation, heterochromatin formation, genomic stability, cell cycle progression, and development through its methyltransferase activity (42). The *KMT5A* variant c.31C > T (NM_020382.7) in exon 2 leading to p.R11C was deemed deleterious with a CADD score of 20.5, and classified as pathogenic according to ACMG criteria PS2, PS4, PM2, and PP3 (Table 2). *KMT2C* was selected as the primary candidate gene due to its significantly higher CADD score of 27 and its established association as a NDD gene.

Within family 5, we identified a sole candidate variant in *SCFD2* (Sec1 family domain containing 2) at 4q12. The homozygous missense variant c.1148C > T (NM_152540.4), p.P383L in the exon 4 was deemed deleterious and probably damaging with a high CADD score of 27 and classified as likely pathogenic based on ACMG classification criteria PS4, PM2, PM3, and PP3 (Table 1). p.P383L variant is located in sec family domain (aa 377-665) and therefore could cause disturbances in the functional properties of SCFD2 protein. Notably, several studies have proposed this gene as a potential susceptibility gene for neurodevelopmental disorders including autism. A genome-wide association study identified *SCFD2* as one of the susceptibility gene among 15 highlighted genes on chromosome 4 for schizophrenia, bipolar disorder, and major depressive disorder (43). Additionally, a comprehensive review listing 792 known or proposed ASD susceptibility genes identified *SCFD2* as a contributor to ASD (44). Interestingly, another report spotlighted *SCFD2* as an autism-implicated gene with high-confidence single nucleotide polymorphisms in its 3’UTR miRNA recognition element, potentially affecting its expression (45).

In family 6, we discovered two *de novo* and two homozygous recessive variants in four different genes. *DHX30* and *PDPR* were found to have *de novo* variants, while *LRRC18* and *PARP14* exhibited autosomal recessive variants. *DHX30*, known as DExH-box helicase 30 (MIM 616423), encodes an RNA-dependent RNA helicase that utilizes ATP hydrolysis to unwind RNA secondary structures, thereby regulating RNA metabolism and function. Previous research identified six different *de novo* heterozygous missense mutations in 12 patients with neurodevelopmental disorders along with severe motor impairment and language delay in *DHX30* at 3p21.31 (46). The splicing variant c.1813-1G > T (NM_014966.4) in this proband was identified as deleterious, supported by its remarkably high CADD score of 33, suggesting a probable disruption of the protein. It was classified as pathogenic by ACMG classification criteria PVS1, PS2, PS4, PM2, PP2, and PP3 (Table 1).

*PARP14*, Poly(ADP-ribose) polymerase family member 14 (MIM 610028), plays a role in poly(ADP-ribosylation), that is an immediate DNA damage-dependent post-translational modification of histones and other nuclear proteins, helping in the survival of injured proliferating cells (47). In exon 6 of PARP14 at 3q21.1, we identified the variant c.2906C > G (NM_017554.3). The variant resulted in a deleterious substitution of p.A969G with a CADD score of 22.6. It was classified as possibly pathogenic by M-CAP and likely pathogenic by ACMG classification of PS4, PM2, PM3, PP3 (Table 2).

*PDPR* (Pyruvate dehydrogenase phosphatase regulatory subunit, MIM 617835) inhibits the activity of the catalytic subunit (PDP1, MIM 605993) in a Mg (2 +)-dependent and Ca (2 +)-stimulated manner (48). In exon 7 of *PDPR* at 16q22.1, we identified the variant c.697G > C (NM_001322118.1), resulting in deleterious p.E233Q with a CADD score of 25.3. According to ACMG classification PS2, PS4, PM2, and PP3, it was classified as pathogenic (Table 2).

*LRRC18* (Leucine rich repeat containing 18, MIM 619002) at 10q11.23, known for its involvement in the regulation of spermatogenesis (49), harbored the variant c.416_417delCAinsTG (NM_001006939.4) in exon 2. It led to a deleterious variant p.T139M with a CADD score of 25.4. Based on ACMG classification (PS4, PM2, PM3, PP3), it was classified as likely pathogenic (Table 2).

To date, *PDPR*, *LRRC18*, and *PARP14* have not been associated with neurodevelopmental disorders. In family 6, *DHX30* was selected as the primary candidate gene over *PARP14*, *PDPR*, and LRRC18 due to its significantly higher CADD score of 33, its classification as pathogenic according to ACMG criteria, and its established association as a NDD gene.

In summary, our study has revealed the involvement of four known genes (*DEAF1*, *CLCN3*, *KMT2C*, and *DHX30*) as well as two novel genes (*TRPC4* and *SCFD2*) in autism spectrum disorder across six families from Qatar.

### 2.2. Molecular modeling of variants in putative candidate genes

**FIGURE 3:**
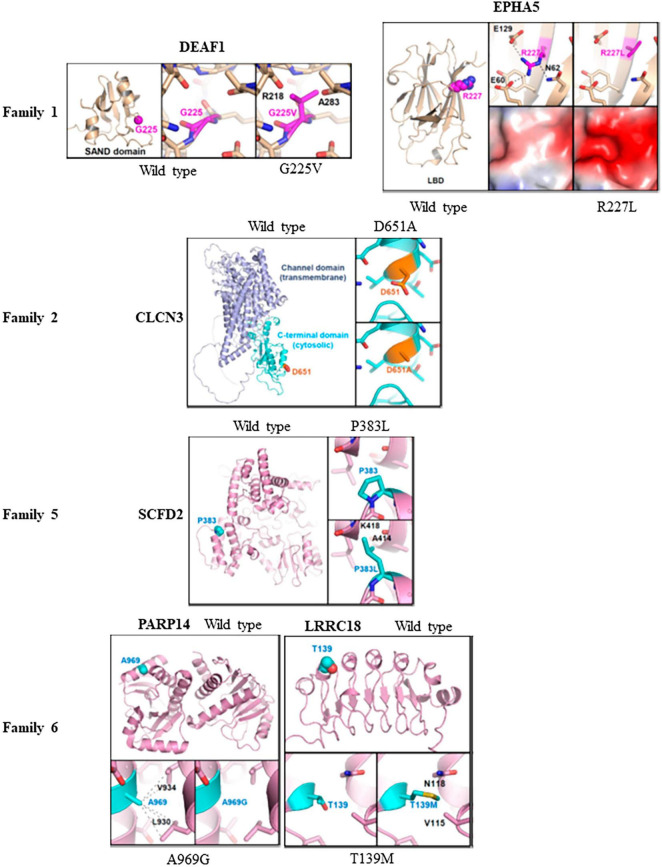
Molecular modeling of missense variants in candidate genes: Molecular modeling was conducted to assess the impact of six missense variants on protein stability and interactions with other proteins. This was done using three-dimensional protein structures of the candidate genes, either obtained from X-ray crystallography (PDB codes: 4ET7 for EPHA5 and 4D86 for PARP14) or constructed by Alphafold-based prediction. For the structures based on Alphafold, we referenced the corresponding entries in the Alphafold protein structure database (https://alphafold.ebi.ac.uk). The wild type of protein structures was compared with the corresponding variant structures using PyMol program. In family 1, the G225V variant in DEAF1 showed severe steric hindrance, indicating impaired protein folding and structural instability compared to the wildtype protein. Additionally, the R227L variant in EPHA5 altered the surface shape and charge of the ligand-binding domain, which might affect its association with putative binding partners. However, R227 is not situated in the ligand-binding surface of the EPHA5 LBD, suggesting that the variant L227 does not disrupt ligand binding. In family 2, the D651A substitution in CLCN3 was found to potentially disturb protein recognition mediated by the C-terminal domain. In family 5, severe steric hindrance was detected in SCFD2-P383L variant leading to impaired protein folding, and structural instability. In family 6, A969G substitution in PARP14 decreases intramolecular hydrophobic interaction, affecting the proper protein folding. Moreover, the LRRC18-T139M variant caused steric hindrance and disrupted the proper protein folding of the leucine-rich repeats and caused structural instability.

The effects of missense variants in ten candidate genes were analyzed at an atomic level using molecular modeling. Homology protein models were employed to examine three-dimensional structural models of various proteins. Crystal structures were utilized for EPHA5 and PARP14, while Alphafold predictions from the website https://alphafold.ebi.ac.uk were used for DEAF1, CLCN3, SCFD2, LRRC18, LRIF1, RGS22, KMT5A, and PDPR. For the DEAF1 variant G225V, the structural modeling based on Alphafold prediction revealed a severe steric hindrance between the side chain of V225 and the main chain carbonyl atoms of Arg218 and Ala283. As a result, the G225V substitution is expected to impair proper protein folding and induce structural instability within the SAND domain (residues 193-273) of DEAF1 (Figure 3, top).

In the crystal structure of EPHA5, Arg227 mediated intramolecular electrostatic interactions with Glu60 and Glu129. Additionally, it formed a hydrogen bond with Asn62. The R227L substitution in EPHA5 induced alterations in the surface shape and charge of the ligand-binding domain (LBD; residues 60–238), which might affect the association of EPHA5 with its putative binding partners. However, it is important to note that Arg227 is not located in the ligand-binding surface of the EPHA5 LBD, indicating that this variant does not appear to interfere with its ligand binding (Figure 3).

In CLCN3 (D651A) modeling, no severe alterations at a molecular level were observed by the D651A substitution in CLCN3. However, since Asp651 is located at the cytosolic C-terminal cystathionine β-synthase (CBS) domain, there is a possibility that the D651A substitution could affect the C-terminal domain-mediated protein recognition of CLCN3 (Figure 3).

Regarding SCFD2 (P383L) modeling, severe steric hindrance was observed between the side chain of L383 and the main chain carbonyl of Ala414, as well as the main chain amide of Lys418 in the Alphafold-based structural model. Consequently, the P383L substitution is expected to impair the proper protein folding, leading to structural instability of the SCFD2 (Figure 3). Analysis of crystal structure of PARP14 indicated that the A969G substitution led to the loss of A969-mediated intramolecular hydrophobic interaction with L930 and V934, which should negatively affect the proper protein folding (Figure 3). In the Alphafold-based structural model of LRRC18 (T139M), steric hindrance was also evident between M139 and V115/N118. Therefore, the T139M substitution is likely to disrupt the proper protein folding of the leucine-rich repeats and cause structural instability of LRRC18 (Figure 3). While protein modeling of the PARP14 with A969G variant and the LRRC18 with T139M variant indicated protein misfolding in family 6, their impact is considered minor compared to the potential protein truncation resulting from the splice variant of DHX30.

Structural modeling of mutations in the disordered loop region is challenging due to their high flexibility. This limitation applies to LRIF1 (I414V), KMT2C (W858X), and KMT5A (R11C). It is worth mentioning that isoleucine and valine share similar structural and biochemical characteristics, and therefore the I414V substitution in LRIF1 is not expected to induce significant molecular-level alteration (Supplementary Figure 1). Furthermore, no significant molecular-level alterations were observed for the A796T substitution in RGS22 and the E233Q substitution in PDPR. It is difficult to interpret their biological consequences at the molecular level. Protein modeling for TRPC4 in family 3 and DHX30 in family 6 was not performed due to potential truncation resulting from splice variants.

### 2.3. Role of interacting protein partners of potential candidate gene variants

In the first family, we identified two potential missense candidate variants, one in *DEAF1* as a *de novo* and another in *EPHA5* as a homozygous variant. *DEAF1* interacts with several genes at the protein level, including *FBXO11*, *DDX58*, *AIMP2*, *GSK3B*, and *HRAS*. They are associated with NDDs including autism, ID, DD, etc. Multiple *de novo* variants in *FBXO11* (F-Box only protein 11) have been found to disrupt its protein expression and localization, leading to neurodevelopmental disorder (NDD) through haploinsufficiency (50). *AIMP2* has been identified as a candidate gene for late-onset Parkinson’s disease, expressed in the mesencephalon, and associated with dysregulation in PD associated pathways (51). Another interacting protein, *HRAS*-germline mutations were involved in Costello syndrome, a multiple congenital anomaly and intellectual disability syndrome (52). Since family 1 is not consanguineous, we chose to give priority to the well-known autosomal dominant neurodevelopmental gene *DEAF1* over *EPHA5*, which has a homozygous variant. This was supported by the results of protein modeling, which revealed a significant steric hindrance caused by the *DEAF1* variant, whereas the *EPHA5* variant did not seem to affect its ligand binding.

In the second family, *CLCN3* emerged as the prime candidate gene over *LRIF1*. *CLCN3* is a member of the CLC family of Cl- channels and Cl-/H + exchangers located in endosomal/lysosomal compartments. Loss of function variants in CLCN3 have been associated with a variety of NDDs such as GDD, ID and dysmorphic features (33). *LARP7*, an interacting protein of *CLCN3* at the protein level, has been reported as a causative of Alazami syndrome characterized by failure to thrive, short stature, and DD symptoms (53). Another interacting protein of *CLCN3*, *SLC35A2* has been linked with a rare type of congenital disorder of glycosylation named as *SLC35A2*-congenital disorders of glycosylation described by neurological impairments with or without skeletal abnormalities (54). Brain somatic *SLC35A2* mutations are also linked to neocortical epilepsy (55). After evaluating factors such as the CADD scores, M-CAP, and ACMG interpretation, the prioritization favors *CLCN3* over *LRIF1*. This choice is supported by protein modeling results, which indicate that the *CLCN3* variant has the potential to impact protein recognition mediated by the C-terminal domain, while the *LRIF* variant is not anticipated to cause substantial molecular-level alterations.

In family 3, we identified *TRPC4* as a prime candidate gene having a *de novo* splice variant. TRPC4, transient receptor potential cation channel, subfamily C, member 4, forms homodimers and heterodimers with other family members such as *TRPC1* and 5 (56, 57). Multiple sporadic variants in TRPC family members associated with NDDs have been reported (58–60). *ITPR1* (inositol 1,4,5-trisphosphate receptor, type 1), an interacting partner of *TRPC4*, is causative for Gillespie syndrome characterized by bilateral iris hypoplasia, congenital hypotonia, non-progressive ataxia, progressive cerebellar atrophy, and intellectual disability as well as autism and schizophrenia (61, 62). *TRPC4* takes precedence as the prime candidate gene over *RGS22* due to its deleterious splicing variant and a high CADD score of 34. This decision was supported by protein modeling results, which indicated no notable molecular-level alterations with the *RGS22* variant.

In family 4, we found two *de novo* missense variants in two lysine methyltransferases, *KMT2C* and *KMT5A*, respectively. The well-known neurodevelopmental gene *KMT2C* interacts with multiple proteins, *WDR5*, *CUL3*, *HCFC1*, and *SETD1A* that are involved in various NDDs. *WDR5* controls neuronal migration and dendrite polarity of pyramidal neurons by affecting reelin signaling (63). *CUL3* has been associated with different NDDs including ASD, DD, and ID (64, 65). *HCFC1* mutations cause intellectual disability by affecting normal brain functioning (66). Dominant *de novo* LoF variants in *SETD1A*, a H3K4 methyltransferase, affect normal cognitive functioning via disbalancing neuronal processes and cause NDDs (67). *KMT2C* was prioritized as the primary candidate gene over *KMT5A* based on its notably high CADD score 27 and its established association as a neurodevelopmental gene.

In family 5, our analysis identified *SCFD2* as the sole candidate gene. According to Biogrid interaction analysis, SCFD2 interacts with multiple protein partners involved in a variety of NDDs. Notably, *PCDHGC4*, one such partner, is a neurodevelopmental gene associated with progressive microcephaly, seizures, and joint anomalies, (68). Furthermore, bi-allelic mutations in *HS2ST1*, another SCFD2 interactor (69), have been associated with developmental delay, intellectual disability, corpus callosum agenesis, facial dysmorphism, and skeletal and renal anomalies (70).

In family 6, we identified one known NDD gene *DHX30* with a *de novo* splice variant as the prime candidate gene. It had 0% gnomAD frequency and a CADD score of 33. *CHD3*, an interacting partner of *DHX30*, is known to cause a neurodevelopmental syndrome with macrocephaly, impaired speech, and language delay (71). Another interacting partner *CHD4* is involved in a multisystemic neurodevelopmental disorder (72). Furthermore, *DHX30* forms an interaction with *CUL4B*, which is associated with X-linked intellectual disability syndrome (73).

### 2.4. Conservation of mutant amino acid in SCFD2 across different vertebrate species

**FIGURE 4:**
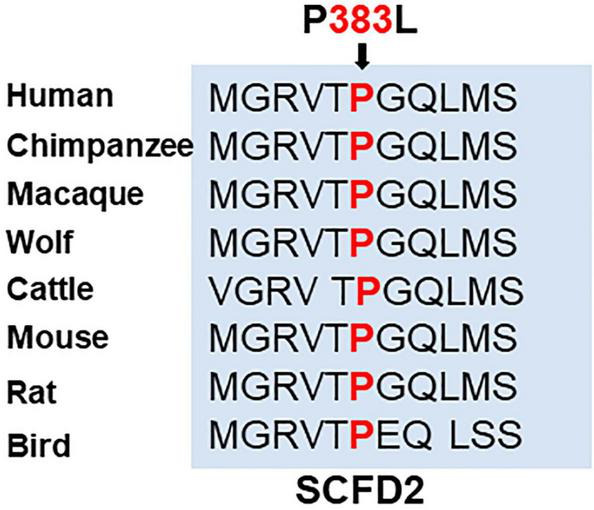
Amino acid conservation in SCFD2 across eight vertebrate species. The sequence alignment for SCFD2 revealed that the P383 residue, mutated in this study, is fully conserved across all eight vertebrate species. The amino acid affected by the missense mutation in our patient is marked in bold red.

To check the importance of the mutant amino acid (P383) in SCFD2, its conservation across eight vertebrate species was checked by multiple alignment. The SCFD2 protein sequence alignment revealed that P383 found mutated in this study was fully conserved among eight vertebrate species namely, human, chimpanzee, macaque, wolf, cattle, mouse, rat and bird. This conservation in protein sequence across eight vertebrate species emphasized the detrimental consequences of this substitution accentuating its vital involvement in shared biological processes across these species (Figure 4).

### 2.5. Digenic model

The subject 4 had a *FMR1* full mutation, being positive for Fragile X-syndrome (FXS) testing (> 200 CGG repeats in the 5′-untranslated region of FMR-1 gene) along with a variant in *KMT2C*, suggesting a putative digenic model (74). There has been a report of concomitant diagnosis of FXS with three other genetic conditions such as Duchenne muscular dystrophy (*DMD*), *PPP2R5D*-related and *MYT1L*-related intellectual disability (74). To date, no prior report has documented the association between an X-linked recessive *FMR1* variant and an autosomal dominant *KMT2C* variant in autism and intellectual disability. With the rising number of cases involving dual genetic diagnoses identified through exome and genome sequencing, it is crucial to recognize the possibility for digenic pattern in the molecular diagnosis of seemingly monogenic disorders.

## 3. Discussion

This study represents one of the first studies conducted on a Middle Eastern cohort, utilizing ES to identify causative genetic variants associated with ASD. Despite ongoing research efforts, the comprehensive understanding of the genetic underpinnings of ASD remains incomplete due to the extensive genetic heterogeneity and the complexity of inheritance pattern. To gain deeper insights into the etiology of ASD through a rigorous correlation strategy involving familial genotyping-phenotyping, it is crucial to explore novel ASD genes in diverse populations.

In this study, we have identified six candidate variants in six independent simplex families in Qatar. These families presented with four known and two novel autosomal dominant heterozygous *de novo* and autosomal recessive candidate genes, with a specific focus on genes associated with ASD. We identified a total of six putative candidate variants, comprising five *de novo* variants and one homozygous variant. Among these variants, four were missense variants, while the remaining two were splice variants. Despite our efforts to prioritize and identify only one primary candidate gene in each proband, most individuals displayed more than one candidate variant. Hence, it is important to recognize that the possibility of the second prioritized gene also playing a role in the phenotype cannot be entirely dismissed. This observation tentatively suggests the potential involvement of a digenic or oligogenic model (75). None of the variants identified in our small cohort were found in the gnomAD and other polymorphism databases. Moreover, these variants exhibited very low frequencies in the Qatar Genome Program (QGP) database, which encompasses a genome sequencing dataset from over 10,000 control population in Qatar. The robustness of our approach was further strengthened by the discovery of variants in genes that had previously been linked to NDDs or ASD (either in high-throughput sequencing databases or in literature).

In families 3 and 5, we identified two novel genes. *TRPC4*, presenting as a splice variant and *SCFD2*, identified as a missense variant. TRPC4 is a member of the TRP superfamily of ion channels, which control ion flow across cell membranes in response to diverse stimuli (57). Particularly, TRPC4 participates in cellular calcium signaling and regulates cation conductance within cells (76). Its main function is to mediate calcium (Ca2 +) influx into cells, forming a non-selective cation channel that enables the movement of calcium ions in response to numerous cellular cues (77). While TRPC4’s specific functions can change based on the cell type and environment, it is notably involved in cell signaling, cardiovascular function and sensory transduction (78–80). Dysregulation of TRPC4 has been linked to multiple pathological conditions. For example, heart conditions including hypertension and cardiac hypertrophy have been linked to abnormal TRPC4 expression (81, 82). Given that calcium signaling can impact cell proliferation and migration, some studies suggest TRPC4’s potential role in cancer progression (83). TRPC4 involvement in pulmonary hypertension is noted, especially in the smooth muscle cells of the pulmonary artery (82, 84). Furthermore, TRPC4 channels in neurons influence synaptic transmission, and dysfunction in these channels may be associated with neurological conditions such as epilepsy (85). On the other hand, SCFD2 is predicted to be involved in vesicular transport processes, particularly concerning vesicle docking and exocytosis. While the exact function of SCFD2 remains elusive, there is some indication of its potential role in carcinogenesis. One research observed p53 binding to the SCFD2 promoter following hypoxia and DNA damage, while another documented decline in breast cancer cell growth *in vitro* upon SCFD2 knockdown (86, 87). Mice with a Scfd2 knockout exhibited phenotypes related to cellular homeostasis, metabolism, and mortality/aging.^1^

In addition to high intolerance to truncating variants (pLI = 1), *CLCN3* is also predicted to be intolerant to missense variants (missense Z score = 4.37 in gnomAD). Thus, the D651A substitution, which potentially disrupts protein recognition mediated by the C-terminal domain, is likely to represent a loss-of-function mutation in *CLCN3* in family 2. Furthermore, the presence of eight different heterozygous *de novo* missense mutations in *CNCL3* among neurodevelopmental patients is likely indicative of loss-of-function mutations, resulting in haploinsufficiency (33). The candidate gene *DHX30* in family 6 has a high constraint for loss-of-function variants in gnomAD (pLI = 1). Thus, the splice acceptor variant identified in this gene in family 6 is likely a deleterious mutation.

Among the three consanguineous simplex families, we discovered only one autosomal recessive gene in family 5, while the other two families exhibited autosomal dominant *de novo* variants. This finding implies that in consanguineous families, it is possible for autosomal dominant or X-linked recessive genes to be involved instead of autosomal recessive genes, especially when only a single family member is affected.

Trio-based ES is known to be a powerful approach for identifying novel ASD candidate genes, indicating the significant contribution of *de novo* mutations to the genetic etiology of non-consanguineous ASD cases (24). In this study, we have identified five *de novo* mutations across six trios, reinforcing the notion that *de novo* mutations contribute substantially to the ASD genetic etiology, even in consanguineous simplex families.

In order to determine their significance in ASD, it is crucial to identify additional ASD or neurodevelopmental individuals with *de novo* mutations. Additionally, performing functional assays to evaluate the impact of the amino acid change on the corresponding protein will be essential in confirming the pathogenicity of these missense variants.

## 4. Conclusion

This study represents one of the early investigations conducted on a Middle Eastern cohort to uncover causative genetic variants associated with ASD. In this study, we identified six candidate variants in independent simplex families in Qatar, involving both known and novel autosomal dominant and autosomal recessive genes associated with ASD. Our findings included *de novo* and homozygous variants, predominantly missense and splice variants. Multiple individuals exhibited more than one candidate variant, suggesting the potential involvement of digenic or oligogenic models. These variants were rarely observed in population databases and showed low frequencies in the control population dataset. Significantly, we have discovered two novel autism genes, *TRPC4* and *SCFD2*. Moreover, the D651A substitution in *CLCN3* and the splice acceptor variant in *DHX30* are likely to be deleterious mutations. Additionally, the presence of multiple *de novo* mutations across trios further emphasizes the significant contribution of *de novo* mutations to ASD’s genetic etiology.

## 5. Materials and methods

### 5.1. Human subjects

#### 5.1.1. Autism diagnosis

Students having suspicions of ASD are sent to the Child Development Center-Hamad Medical Corporation (CDC-HMC) or the Shafallah Center for a full medical and diagnostic evaluation. Both centers have ASD diagnosis teams, comprising developmental pediatricians, psychologists, general pediatricians, speech pathologists, behavioral analysts, and speech therapists. To diagnose whether the children have ASD, trained diagnostic team utilizes Autism Diagnostic Interview—Revised (ADI-R) (88) and the Autism Diagnostic Observational Schedule (ADOS) (89). Multilingual professionals conduct assessments in either Arabic or English, and where necessary, other languages are facilitated with translators, depending on family’s preference. The research focused on children aged 5–12 residing in Qatar, encompassing both Qatari and expatriates’ population, during the recruitment and sample collection phase.

#### 5.1.2. Family 1

The subject 1 is a 16-year-old Arabic female from Tunisia with a history of autism, loss of acquired language, and social skills with aggressive behaviors. The parents were of 47 years (mother) and 53 years (father) old at the time of the birth of the subject. Family had no reported history of consanguinity. The subject was born via a normal, full-term pregnancy, delivered by C-section as the second child. The C-section was performed as an emergency due to complications arising from the umbilical cord being wrapped around the neck of the baby. Otherwise, there were no prenatal, or postnatal complications. The subject achieved normal milestones, sitting at 6 months, and walking at 12 months. There were no issues with the loss of acquired motor skills. The subject started babbling and uttering words such as “papa” and “mama,” as well as two-word sentences, within the expected time frame. However, at the age of 30 months, there was a regression in acquired language and social skills. There were no issues of seizure, head trauma, serious childhood illnesses, or allergies in the subject. Aggressive behavioral problems also emerged at the age of 30 months. The subject underwent an ADI (autism diagnostic interview) to confirm the autism diagnosis. Fragile X-syndrome and Rett syndrome were ruled out through normal test results. EEG was normal and the subject did not undergo metabolic screening, MRI, or CT-scan. There is no history of any neuro developmental symptoms in the family.

#### 5.1.3. Family 2

The subject 2 is a 30-year-old Qatari female with a history of autism and learning problems. She was born through a normal, full-term (38 weeks) vaginal delivery, as the second child of her late father (60 years old) and her mother (27 years old). There was reported family history of consanguinity. The subject’s birth did not involve any delivery or postnatal complications. Her developmental milestones were achieved within the expected timeframe. She started sitting independently at 6 months and began walking at 12 months, indicating no issues with the acquisition of language or motor skills. The primary spoken language at home was Arabic. Although the subject started babbling at the age of 2 months, her progression of saying “baba,” “mama,” one-word and two-word sentences was delayed until 96 months (8 years). In fact, she still struggles to speak in two-word sentences. It is noteworthy that she belongs to a consanguineous family, and on the father’s side, three cousins had language delay and learning problems. However, the subject had no behavioral problems. There is no record of seizures, head trauma, or serious childhood illnesses in the subject’s medical history. Fragile X-syndrome and Rett syndrome tests yielded normal results. The subject underwent a normal MRI/CT scan at the age of 60 months, and metabolic screening and EEG were not conducted.

#### 5.1.4. Family 3

The subject 3 is a 16-year-old Qatari female with a history of autism, chronic lung disease, and global developmental delay. The subject is an offspring of 50 -year-old father and 42-year-old mother. The subject was born prematurely at 25 weeks through a normal vaginal delivery, with an APGAR score of 4 and 9 at one and 5 min, respectively. Unfortunately, the subject’s twin brother developed severe respiratory stress and died only after 3 h. The subject herself had intraventricular hemorrhage and hydrocephalus, which necessitated the placement of a ventriculoperitoneal shunt. As a result, she has delayed development and cortical blindness. MRI scans revealed ventriculomegaly, widened cisterns, and hypoplastic cerebellar hemispheres. The subject belongs to a non consanguineous family. During the initial months of the subject’s life, several medical conditions were observed. At 1 month, severe bilateral ventriculomegaly, moderate third ventricular dilatation, and suspected mild fourth ventricular dilatation were observed. At 2 months, moderate to severe hydrocephalus, thinning of the cerebral mantle, hemosiderin deposits, and subacute blood products were found. Poor visualization of the cerebellar hemispheres was noted. Follow-up at 4 months showed persistent hydrocephalus, destruction of the left cerebellar hemisphere, and hyperdense areas in the posterior fossa. Minimal extracerebral cerebrospinal fluid space prominence was noted. At around 6 months, the subject displayed characteristic symptoms such as wide and bulging anterior fontanel, sunset appearance of the eyes, and increased head circumference. At 13 months, the subject had a history of chronic lung disease and global development delay. Mild airway obstruction was observed, but the lung volume was normal with no significant air trapping. A radiograph at 2 years showed normal bones and joints, with the VP shunt tube visible in the lower abdomen. In summary, the subject has a complex neurological condition with hydrocephalus, ventriculomegaly, and potential cerebellar abnormalities. The subject exhibited normal bone and joint health.

#### 5.1.5. Family 4

The subject 4 is a 14-year-old Qatari male with symptoms of autism, learning and speech delay. The subject is offspring of a 49-year-old father and a 42-year-old mother. He was born through normal vaginal delivery resulting from full-term pregnancy (38 weeks) without complications. Developmental milestones such as independent sitting at 7 months and walking at 15 months were achieved on time. However, the subject exhibited delayed language and speech skills, including babbling at 12 months, saying “baba” and “mama” at 36 months, and speaking single words only at 36 months. The formation of two-word sentences were also delayed, and the subject continues to struggle with complex sentence formulation. Arabic is the spoken language at home. Additionally, the subject had learning difficulties, intellectual disability, and tested positive for Fragile X-syndrome with more than 200 CGG repeats in the 5′-untranslated region of *FMR-1* gene. The subject does not belong to a consanguineous family. On the maternal side, one uncle had speech and learning delays, while another uncle had learning problems. On the paternal side, an uncle had intellectual disability. There is no report of seizures, head trauma, and serious childhood illnesses. The subject was diagnosed with ASD at 42 months of the age.

#### 5.1.6. Family 5

The subject 5 is a 14-year-old Qatari male with symptoms of autism, language delay, intellectual disability, and loss of acquired skills. He belongs to a consanguineous family, with his father being 56 years old at the time of his birth. The subject was born through normal vaginal delivery at full term (38 weeks) as the fifth child. While there were some pre-natal complications (GDM), there was no delivery, neonatal or postnatal complications. The subject achieved independent sitting and walking milestones later than expected, at 17 months. Moreover, the subject displayed signs of skill regression at an early age of 18 months. Regarding language and speech development, the subject displayed delayed babbling at 7 months, saying “baba” and “mama” at 12 months, and producing single words only at 10 months. Arabic was the spoken language at home. Despite initially meeting the milestone of speaking single words on time at 10 months, there was subsequent language delay and a lack of acquired motor skills and acquired language skills at 18 months. Family history reveals language delay on the mother’s side and cousins with intellectual disability on both the mother and father’s sides. While there are no reports of seizures or serious childhood illnesses, but of the subject experienced a head trauma incident at the age of 8 months. Fragile X-syndrome molecular testing yielded negative results.

#### 5.1.7. Family 6

The subject 6 is a 18-year-old male having symptoms of autism, learning and speech delay. He is the offspring of a 52-year-old father, and the family has a Sudanese lineage with parents who are first cousins and consanguineous. The subject was born through assisted vaginal delivery as a first child, without complications during delivery. However, the newborn experienced neonatal complications, specifically fetal hypoxia, requiring a 24-h stay in the neonatal intensive care. Milestones-wise, the subject achieved independent sitting at 6 months on time, but walking was delayed until 18 months. Arabic is the spoken language at home, and while the subject started babbling on time, there was delay in saying “baba,” “mama,” single words, and double words at 24 months. The subject had no problem in loss of acquired motor skills and loss of acquired language skills. The subject had behavioral issues, and displaying withdrawn behavior at home and in public, but there was no loss of acquired social skills. There are no reported neurodevelopmental issues on the father or mother’s side of the family, except for one of the mother’s brothers having eye issues. The subject has no history of seizures, head trauma, serious childhood diseases, or allergies, although anemia with iron deficiency was reported at an early age.

### 5.2. Exome sequencing (methods)

ES, a cost-effective alternative to GS, detects variants in the coding regions of the genome, known as the exome, which harbor up to 85% disease-causing mutations. The workflow of ES involves isolating genomic DNA from blood samples obtained from affected individuals and their parents as controls. The isolated DNA is then used to prepare exome-enriched libraries using AgilentSureSelectXT kit. The libraries, after enrichment, are pooled into 16 different pools and clustered on a cBot platform. Subsequently, the samples are sequenced on an Illumina HiSeq 4000 using paired end reads (2 bp × 150 bp) at a coverage of 30X per sample. The obtained next generation sequencing data is aligned to the human genome reference sequence, (hg19) and variant are called using the CLC genomics software from Qiagen. Finally, clinically relevant variants are annotated using QC-IT from Qiagen. In this study, we analyzed the ES data obtained from six syndromic autism trios (affected child and both non-affected parents) from Qatar.

Following the manufacturer’s recommendations, the QIAamp DNA blood kit (Qiagen, Hilden, Germany) was used to isolate DNA from peripheral blood samples collected from each participant. Fragile X screening was performed on each patient, and patients with *FMR1* gene change were excluded from all future genetic analyses and phenotypic cluster analysis. This exclusion was necessary because Fragile-X syndrome is a well-known cause of syndromic autism and intellectual disability and has a distinct molecular pathomechanism.

### 5.3. Identification of variants

We employed a combination of several techniques to assess the pathogenicity of variants identified in the VCF files. The initial list of candidate variants was obtained by implementing the following filtering steps: variants with a frequency below 0.1% in ExAC/gnomAD v2.11/1000g2015 were included; only exonic, splicing, non-synonymous, and stopgain variants with a sufficient coverage were considered; variants predicted to be benign or tolerated in PolyPhen and SIFT were discarded, while those with CADD score ≥20 were retained; variants present in more than 1% of in-house controls (i.e., individuals without ASD or any neuropsychiatric disease) were excluded. Finally, M-CAP analysis also indicated likely pathogenicity of the identified variants (31). Furthermore, we performed additional filtering based on annotation data from public databases such as the Mouse Genome Database (MGD), OMIM, PubMed, and ClinVar. The final section of variants was guided by ACMG guidelines, absence in control datasets (gnomAD and QGP), relevance of interacting proteins in neurodevelopmental disorders and autism based on literature, presence of sporadic variants in the Human Gene Mutation Database (HGMD), expression patterns in human tissues, and phenotypes observed in available knock-out/deletion model organisms. Protein modeling is employed to assess the impact of missense variants on the structure and functionality of our candidate genes identified. By following this rigorous procedure, we obtained a final list of six candidate variants, including five *de novo* and one recessively inherited.

### 5.4. Protein modeling

Molecular modeling of potential candidate genetic variants was conducted using the three-dimensional protein structures obtained from X-ray crystallography [(PDB codes: 4ET7 for EPHA5 and 4D86 for PARP14) or constructed by Alphafold-based prediction]. For the Alphafold protein structures, the corresponding entries in the Alphafold protein structure database^2^ were used: O75398 for DEAF1, P51790 for CLCN3, Q5T3J3 for LRIF1, Q8NE09 for RGS22, Q2YDW7 for KMT5A, Q8WU76 for SCFD2, A8MT40 for PDPR, and Q66HD6 for LRRC18. However, KMT2C was not registered in the database, so the location of its Trp858 in the disordered loop region was predicted based on information from the Uniprot database.^3^ To introduce the variants into the protein structures and generate visual representations, we utilized the PyMOL program.^4^

## Data availability statement

The data presented in the study are deposited in the Clinvar repository, with accession numbers SCV004009700, SCV004009701, SCV004009702, SCV004009703, SCV004009704, and SCV004009705 for the variants reported in Table 1. The variants reported in Table 2 have been submitted to ClinVar repository with submission IDs SUB13902132, SUB13902145, SUB13902147, SUB13902149, SUB13902155, SUB13902158, and SUB13902162.

## Ethics statement

This study was approved by the Institutional Review Board of QBRI-2010-002- “Study of Genetic and Environmental Etiologic Factors in Autism.” The studies were conducted in accordance with the local legislation and institutional requirements. Written informed consent for participation in this study was provided by the participants’ legal guardians/next of kin.

## Author contributions

VG, AB-M, AY, and FA: data curation. VG, AB-M, BK, DV, ZJ, and AK: formal analysis. H-GK: funding acquisition and project administration. VG and AB-M: investigation, methodology, validation, and writing – original draft. FA: resources. VG, AB-M, BK, DV, ZJ, AK, and PJ: software. PJ and H-GK: supervision. VG, AB-M, LS, PJ, LL, and H-GK: writing – review and editing. All authors have reviewed and approved the final manuscript.

## Footnotes

1
https://www.informatics.jax.org/marker/MGI:2443446

2
https://alphafold.ebi.ac.uk

3
https://www.uniprot.org/uniprotkb/Q8NEZ4/entry

4
http://www.pymol.org
