# Supplementary material for: Identification of two novel autism genes, TRPC4 and SCFD2, in Qatar simplex families through exome sequencing
Source: Front Psychiatry. 2023 Oct 31;14:1251884. doi: 10.3389/fpsyt.2023.1251884 (PMC10644705; doi:10.3389/fpsyt.2023.1251884)
Supplement: Supplementary file 1 [file Data_Sheet_1.PDF]

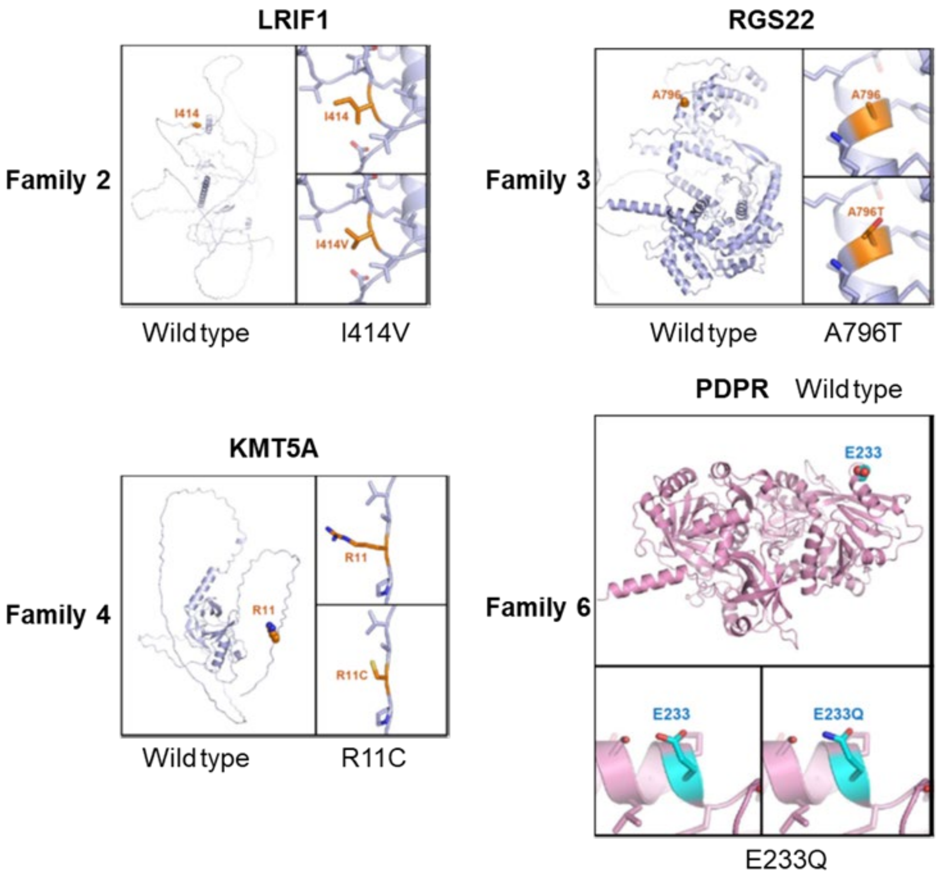


**Suppl. Figure 1: Molecular modeling of missense variants in candidate genes:** Molecular modeling was conducted to assess the impact of four missense variants on protein stability and protein-protein interactions. The wildtype protein structures were compared to the corresponding structures of protein variants. All figures were generated using the PyMol program. In family 2, LRIF1-I414V variant and in family 4, KMT5A-R11C variant were in the disordered loop region that is not generally targeted for structural modeling due to their high flexibility. In family 3, A796T substitution in RGS22 and in family 6, E233Q substitution in PDPR did not exhibit significant molecular-level alterations.
